# Centrosome proteins form an insoluble perinuclear matrix during muscle cell differentiation

**DOI:** 10.1186/1471-2121-10-28

**Published:** 2009-04-21

**Authors:** Vlastimil Srsen, Xavier Fant, Rebecca Heald, Catherine Rabouille, Andreas Merdes

**Affiliations:** 1 Wellcome Trust Centre for Cell Biology, University of Edinburgh, King's Buildings, Edinburgh EH9 3JR, UK; 2 University of California, Berkeley, Department of Molecular & Cell Biology, 142 Life Sciences Addition # 3200, Berkeley, CA 94720-3200, USA; 3 The Cell Microscopy Centre, Department of Cell Biology and Institute of Biomembranes, University Medical Centre Utrecht, 3584CX Utrecht, The Netherlands; 4 Centre National de la Recherche Scientifique-Pierre Fabre, UMR 2587, 3 rue des Satellites, 31400 Toulouse, France

## Abstract

**Background:**

Muscle fibres are formed by elongation and fusion of myoblasts into myotubes. During this differentiation process, the cytoskeleton is reorganized, and proteins of the centrosome re-localize to the surface of the nucleus. The exact timing of this event, and the underlying molecular mechanisms are still poorly understood.

**Results:**

We performed studies on mouse myoblast cell lines that were induced to differentiate in culture, to characterize the early events of centrosome protein re-localization. We demonstrate that this re-localization occurs already at the single cell stage, prior to fusion into myotubes. Centrosome proteins that accumulate at the nuclear surface form an insoluble matrix that can be reversibly disassembled if isolated nuclei are exposed to mitotic cytoplasm from Xenopus egg extract. Our microscopy data suggest that this perinuclear matrix of centrosome proteins consists of a system of interconnected fibrils.

**Conclusion:**

Our data provide new insights into the reorganization of centrosome proteins during muscular differentiation, at the structural and biochemical level. Because we observe that centrosome protein re-localization occurs early during differentiation, we believe that it is of functional importance for the reorganization of the cytoskeleton in the differentiation process.

## Background

The formation of muscle during embryonic development involves the differentiation of myoblasts into long, fibrous cells. In this differentiation process, myoblasts withdraw from the cell cycle and fuse into multinucleate, syncytial myotubes [1]. The microtubule cytoskeleton is reorganized from a radial network into a parallel array of filaments aligned along the long axis of the cells [2]. It is believed that this reorganization is a prerequisite for the elongation and fusion of myoblasts, and for the subsequent alignment and organization of sarcomeres [3-7]. Microtubule reorganization is paralleled by reorganization of centrosomal proteins: myoblasts possess a morphologically recognizable centrosome with characteristic marker proteins concentrated in the pericentriolar material, whereas myotubes show perinuclear localization of a multitude of centrosome proteins [8-10]. Consequently, polymerization of microtubules is initiated in part from the surface of the nucleus [8,10,11]. It has been reported that reorganization of microtubules and relocalization of centrosome proteins to the nuclear surface occurs after fusion of myoblasts into myotubes [8]. However, the precise kinetics of this reorganization are unknown. Morover, it is unknown how centrosome proteins are attached to the nuclear surface, and how they are organized at the ultrastructural level.

## Results and Discussion

**Figure 1:**
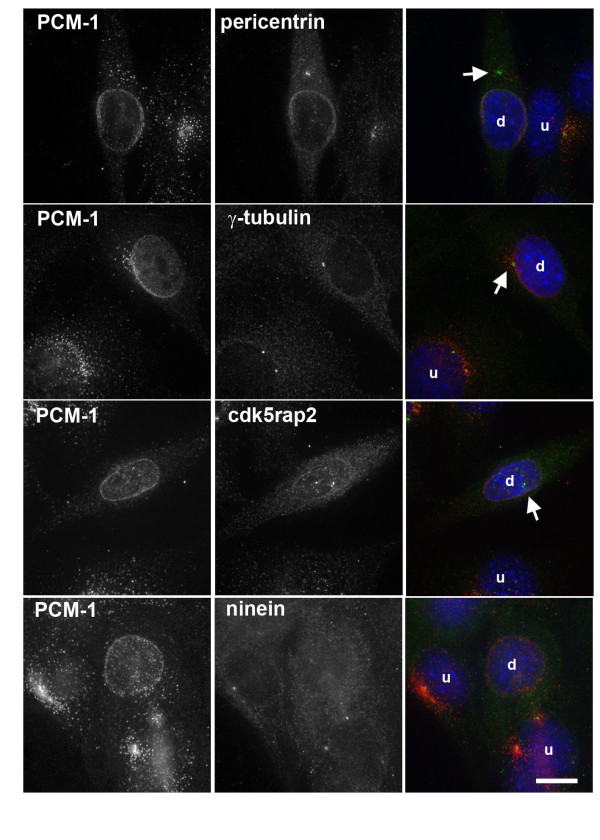
**Relocalization of centrosome proteins to the nuclear surface in differentiating myoblasts**. Cultures of C2C12 myoblasts were induced to differentiate by serum starvation for one day. At this stage, the culture contains undifferentiated myoblasts (u), as well as cells that started to differentiate (d). Immunofluorescence of these cultures was performed to visualize the centrosome proteins PCM-1 (red), as well as pericentrin, gamma-tubulin, cdk5rap2, and ninein (all green). DNA was stained in blue. Arrows indicate partial staining of the remnant centrosomes by pericentrin, gamma-tubulin, and cdk5rap2. Bar, 10 μm.

In order to determine at what stage of the differentiation process relocalization of centrosome proteins occurs, we performed cell culture of mouse C2C12 myoblasts and triggered differentiation by serum withdrawal. Alternatively, we used *H-2K*^b^-tsA58 mouse myoblasts, carrying a thermolabile T-antigen and allowing differentiation upon temperature shift from 33 to 37°C [11,12]. In the undifferentiated state, the centrosomal proteins of both cell lines were found in a single focus within the pericentriolar material adjacent to the nucleus, while the protein PCM-1 ("pericentriolar material protein 1") localized to multiple 'centriolar satellites', as described by [13] and [14]. One day after differentiation of myoblasts was triggered, we observed centrosome proteins at the nuclear periphery already at the single cell stage, prior to fusion into myotubes (Fig. 1).

We investigated the pattern of various proteins and found that significant amounts of pericentrin and PCM-1 accumulated at the nuclear periphery, whereas the centrosome protein cdk5rap2 showed partial relocalization. Only minor amounts of gamma-tubulin were found at the nuclear periphery, consistent with data from [10], and the protein ninein was not found to relocalize to the nuclear periphery in our experiments.

**Figure 2:**
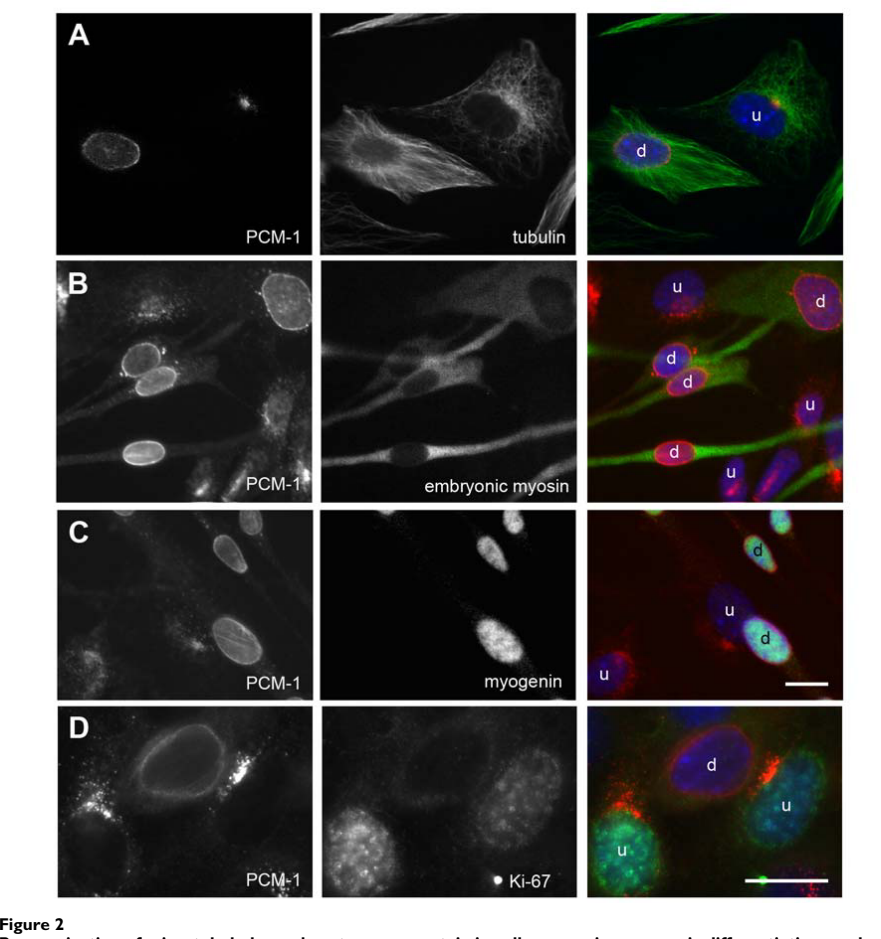
**Reorganization of microtububules and centrosome protein in cells expressing myogenic differentiation markers**. Culture of mouse myoblasts containing undifferentiated (u) cells, and cells that started to differentiate (d). The centrosome protein PCM-1 is stained in red, DNA is stained in blue. In green is marked (A) tubulin, (B) the differentiation marker 'embryonic myosin', (C) the differentiation marker myogenin, (D) the proliferation marker Ki-67. Bars, 10 μm. Identical magnifications in A-C.

A closer look at differentiating C2C12 cells revealed that at the single cell stage, proteins such as pericentrin and PCM-1 started to accumulate at the nuclear periphery in the proximity of the centrosome, whereas areas of the nuclear envelope distal from the centrosome showed less centrosome protein enrichment (Fig. 1, first two rows). At the same time, remnant pericentrin and cdk5rap2 were still visible at the centrosome, and PCM-1 was still partly localized in pericentriolar satellites (Fig. 1). The early relocalization of these proteins from the centrosome to nearby sites of the nuclear surface raises the question as to whether direct transfer from the centrosome is involved, or whether the proteins are recruited to the nuclear surface from a soluble cytoplasmic pool. Both scenarios seem possible: direct transfer from the centrosome to the nucleus might occur by diffusion. Alternatively, at the onset of differentiation, proteins such as pericentrin might be recruited from a soluble pool and localize to the nuclear surface next to the centrosome due to minus-end directed transport along microtubules [15], since the microtubule-organizing centre in these myoblasts is strongly focused at the centrosome and would thus favour deposit of pericentrin in the surrounding area (Fig. 2A, cell marked with 'u'). It is noteworthy, however, that already at the single cell stage microtubules were reorganized into a sun-like array radiating from the nuclear periphery within those cells displaying perinuclear relocalization of centrosome proteins (Fig. 2A, cell marked with 'd').

**Figure 3:**
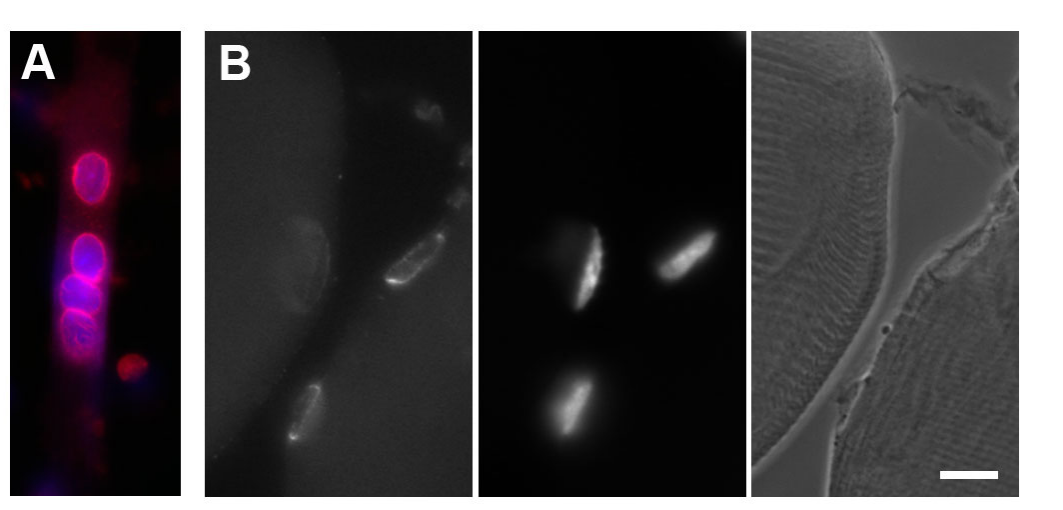
**Perinuclear localization of centrosome proteins persists after fusion of myoblasts into myotubes, and in adult mouse muscle**. (A) Myotube of fused C2C12 cells in culture. Immunofluorescence of PCM-1 (red); DNA is stained in blue. (B) Cryosection through leg muscle from a mouse. Left: immunofluorescence of PCM-1. Middle: nuclei, stained with the DNA marker DAPI. Right: phase contrast image, showing the striations of the muscle tissue. Bar, 10 μm.

To verify that these cells had indeed entered the differentiation programme, we performed immunofluorescence microscopy with differentiation markers. We noticed that all cells in which the centrosome protein PCM-1 had relocalized to the nuclear surface showed cytoplasmic expression of embryonic myosin, an isoform of myosin that is specifically expressed upon onset of muscle cell differentiation (Fig. 2B). Furthermore, these cells expressed the differentiation marker myogenin, a transcription factor that localizes to the nucleus of differentiating cells (Fig. 2C). This marker was absent from cells showing pericentriolar PCM-1 staining. Conversely, these cells with pericentriolar PCM-1 were the only ones that stained positively for the proliferation marker Ki-67 in the nucleus, whereas nuclei in cells with relocalized PCM-1 did not contain Ki-67 (Fig. 2D). Once relocalization of PCM-1 occurred, it persisted until the final stages of differentiation: We detected PCM-1 around the nuclear surface after fusion of multiple C2C12 cells into myotubes (Fig. 3A). Moreover, nuclei in muscle from adult mice showed comparable staining of PCM-1 at their surface (Fig. 3B).

**Figure 4:**
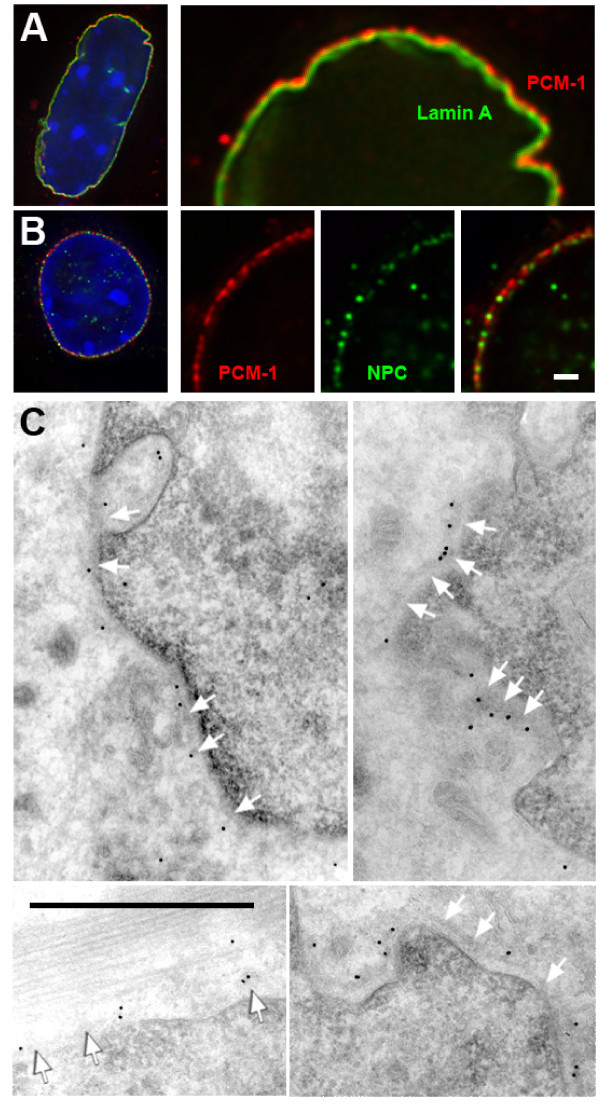
**The centrosome protein PCM-1 localizes to dense structures on the cytoplasmic site of the nuclear envelope**. (A) Deconvolved image of a nucleus from a differentiated *H-2K*^b^-tsA58 cell, expressing GFP-lamin A (green), and stained for PCM-1 (red) and DNA (blue). (B) Nucleus from a differentiated *H-2K*^b^-tsA58 cell, stained for PCM-1 (red), and for nuclear pore complex proteins (NPC, green). Selected areas of the nuclei in (A) and (B) are shown enlarged on the right. (C) Immuno-electron microscopy of cryosections of differentiated C2C12 cells. Different views of cross-sections of the nucleus are shown. PCM-1 is labelled with antibody and protein A, coupled to 10 nm gold. White arrows indicate the outline of a layer of electron-dense material at the outer nuclear surface. Bars in (B) and (C), 1 μm.

We next sought to determine whether centrosome proteins that had assembled around the nucleus were bound at the inside or at the outside of the nuclear envelope. For this purpose, we transfected mouse myoblasts with GFP-tagged lamin A, a protein of the inner nuclear envelope. Deconvolution microscopy revealed that the centrosome protein PCM-1 localized in a rim slightly outside of lamin A (Fig. 4A), suggesting that PCM-1 associates with the outer surface of the nuclear envelope. Because the PCM-1 staining at the nuclear surface was visible in small clusters, we tested whether these sites co-localized with nuclear pores. However, double immunofluorescence with an antibody against a family of nuclear pore complex proteins indicated that most of the PCM-1 clusters were distinct from nuclear pores (Fig. 4B). To reveal further structural details, we performed immuno-electron microscopy of ultra-thin cryosections of differentiated C2C12 cells. PCM-1 was detected with antibodies and gold-coupled protein A. This technique preserved cellular membranes and showed that PCM-1 localized mainly outside the nucleus, away from the outer nuclear membrane (Fig. 4C). Consistent with our immunofluorescence data, immunogold labelling of PCM-1 was often seen in clusters (Fig. 4C, top right). The gold was mostly seen along grey electron-dense material surrounding the nuclear surface (Fig. 4C, arrows). This electron-dense material had a thickness between 30 and 40 nm, suggesting that was part of a tight matrix.

**Figure 5:**
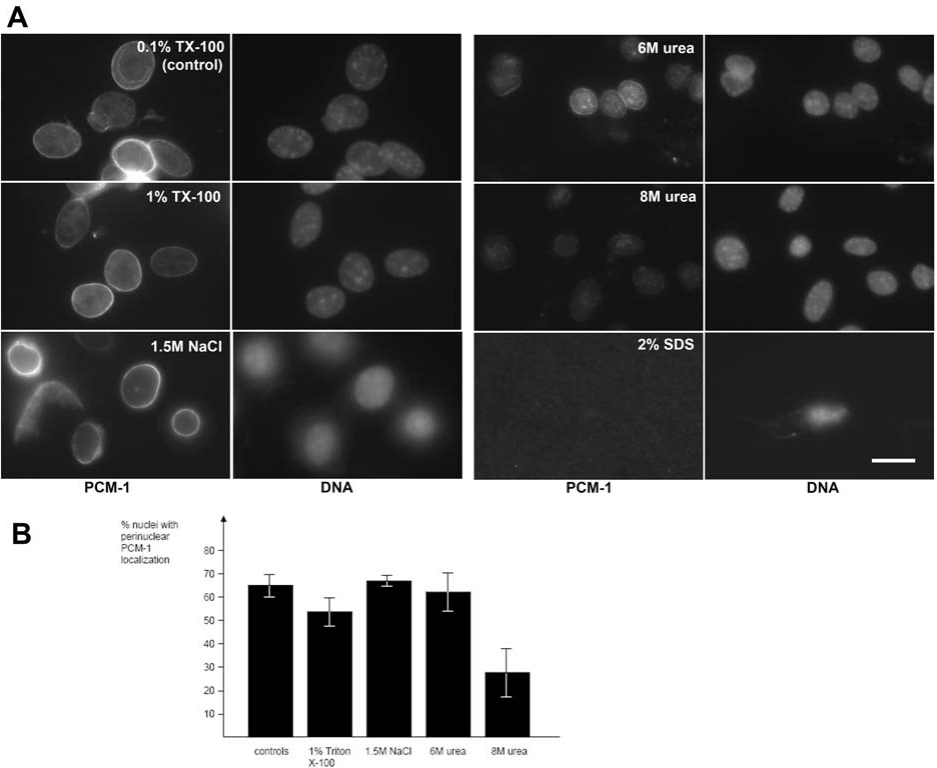
**The centrosome protein PCM-1 is part of a detergent-resistant perinuclear matrix in differentiated muscle cells**. (A) Purified nuclei from differentiated C2C12 cells were incubated for 10 minutes in buffer, containing as indicated, 0.1% Triton X-100, or 1% Triton X-100, or 1.5 M NaCl, or 6 M urea, or 8 M urea, or 2% sodium dodecyl sulfate. For details, see 'Methods'. Nuclei were spun onto coverslips and processed for immunofluorescence of PCM-1. DNA was stained with 4',6-diamidino-2-phenylindole. Bar, 10 μm. (B) Percentage of nuclei that stained positively for perinuclear PCM-1, following treatment as specified in (A). A minimum of three experiments were performed, counting more than 100 nuclei per experiment.

To test for the biochemical behaviour of perinuclear PCM-1-containing material, we purified nuclei from differentiated C2C12 cells that had fused into myotubes. These were separated from non-differentiated myoblasts by short treatment of cultures with trypsin, leading to selective enrichment of the differentiated cells. Following fractionation of cells, we performed extraction of purified nuclei with buffer containing various concentrations of salt, detergents, or urea. We found that perinuclear PCM-1 localization was largely resistant to 1.5 M NaCl, to treatment with 1% Triton X-100, or even to extraction with 6 M urea (Fig. 5A, B). However, PCM-1 was efficiently removed from the nuclear surface in 8 M urea, or in buffer containing the denaturing detergent SDS (sodium dodecyl sulfate) (Fig. 5A, B). Nuclei treated with SDS lost their integrity even after short times of treatment, and could therefore not be quantified reproducibly. Altogether, these results indicate that PCM-1 is part of an insoluble matrix in differentiated muscle cells. Taking into account that several centrosome proteins form a fibrous meshwork surrounding the centrioles in undifferentiated cells [16], it seems plausible to assume that a similar fibrous meshwork forms around the nucleus during differentiation. Proteins such as pericentrin that have large predicted alpha-helical domains, and the potential to form coiled-coil interactions, might contribute to the formation of such a meshwork.

**Figure 6:**
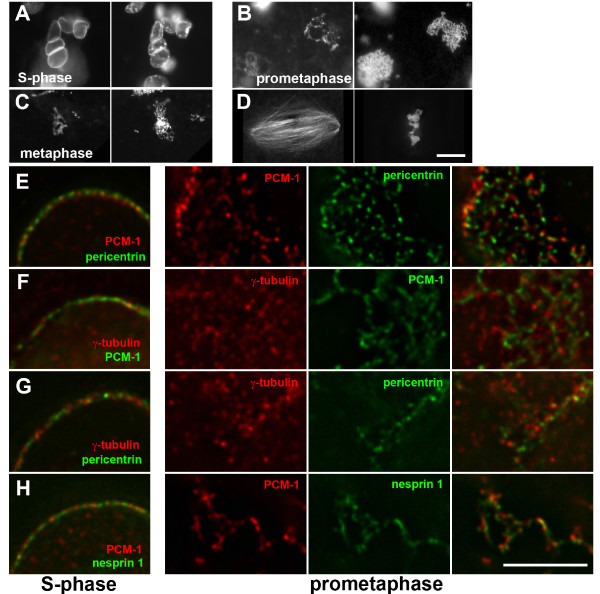
**The perinuclear matrix of centrosome proteins disassembles in mitotic egg extract**. Purified nuclei from differentiated C2C12 cells were incubated in Xenopus egg extract and driven through the cell cycle in vitro. (A) Nuclei in extract in S-phase. Left: PCM-1; right: DNA. Continued incubation of nuclei in extract entering prometaphase (B), and metaphase (C). Condensation of chromosomes (right) and disintegration of the perinuclear matrix of PCM-1 (left) are visible. (D) Incubation of Xenopus sperm in parallel extract reactions produced mitotic spindles. Left: Rhodamine-labelled tubulin; right: DNA. (E – H) Nuclei from differentiated C2C12 cells were incubated in Xenopus egg extract as in (B). Samples in S-phase and prometaphase were processed for immunofluorescence of PCM-1, pericentrin, gamma-tubulin, and nesprin 1, as indicated. Deconvolved sections are shown. Left column: S-phase; second and third column: prometaphase; right column: merged signals of second and third columns. Bars, (D) 10 μm, (H) 5 μm.

To further characterize the nature of the perinuclear meshwork of centrosome proteins, we designed an in-vitro-approach to determine whether its formation was reversible in differentiated C2C12 cells upon re-entry into the cell cycle. Because muscle cells become postmitotic after differentiation, as shown by the lack of Ki-67 staining (Fig. 2D), cultures of differentiated C2C12 cells could not simply be driven into mitosis. To circumvent this problem, we isolated nuclei from differentiated cells and incubated them in cycling egg extracts from Xenopus laevis (Fig. 6, A–C). These extracts are capable of mimicking cell cycle events such as S-phase and mitosis in vitro, and assemble mitotic spindles around various sources of DNA, including exogenously added nuclei (Fig. 6D).

We noticed that upon entry of these extracts into a prometaphase-like state, C2C12 nuclei began to disassemble. DNA condensed into mitotic chromosomes, and the perinuclear 'matrix' of PCM-1 disintegrated into a system of interwoven fibres, and finally disassembled into protein aggregates of varying sizes (Fig. 6B, C). We performed deconvolution microscopy on nuclei, stained with various markers, in S-phase and prometaphase. We found that PCM-1, pericentrin, gamma-tubulin, as well as the nuclear envelope protein nesprin 1 localized to patches on the nuclear envelope that were closely apposed, but without fully co-localizing with each other (Fig. 6, E–H, left column). In samples that had entered prometaphase, we found that PCM-1, pericentrin, and nesprin 1 localized to spots and interconnected fibres that co-localized partly, whereas gamma-tubulin was found more diffusely distributed (Fig. 6, E–H). We therefore believe that PCM-1 and pericentrin form distinct fibrillar structures at the outer nuclear surface that are connected at various contact sites, thus constituting a tight matrix. This matrix may be structurally equivalent to the fibrous pericentriolar material in undifferentiated cells, and it can be disassembled or at least loosened upon entry into mitosis. Consistently, many centrosome proteins, including PCM-1 and pericentrin, are seen in undifferentiated cells during mitosis in a wide crescent-shaped area at the spindle poles or diffuse in the cytoplasm, whereas in interphase they are more focused at the centrosome [14,17,18].

## Conclusion

In this manuscript, we demonstrate that relocalization of centrosome proteins to the nuclear surface is an early event during the differentiation of myoblasts, which occurs prior to their fusion into myotubes. This may imply that the relocalization is important for subsequent differentiation events, for example by affecting microtubule organization. Further, we show that centrosome proteins form a filamentous matrix around the outer surface of the nuclear envelope. Our biochemical experiments demonstrate that this matrix is highly insoluble, but disassembles in mitotic cytoplasm. The assembly and disassembly characteristics of this matrix may provide general insights into the organization of the pericentriolar material of centrosome proteins. Future work will be needed to determine the molecular mechanisms that lead to re-localization of centrosome proteins to the nuclear surface upon muscular differentiation.

## Methods

### Cell culture

C2C12 cells were grown in Dulbecco's modified Eagle's medium, containing 0.5% chicken embryonic extract and 20% fetal calf serum. Differentiation was induced by serum starvation for one or more days, by replacing the regular growth medium with Dulbecco's modified Eagle's medium containing 5% horse serum. *H-2K*^b^-tsA58 myoblasts were cultured and differentiated as described [11]. Transfections of *H-2K*^b^-tsA58 cells with GFP-tagged lamin A [19] were performed using Lipofectamine Plus transfection agent (Invitrogen).

### Microscopy

Cells were grown on glass coverslips and fixed in methanol at -20°C for 10 minutes. Immunofluorescence was performed using standard procedures. Antibodies used in this study were against PCM-1 (rabbit and mouse anti-PCM-1, [14]), pericentrin (rabbit anti-pericentrin, Covance), gamma-tubulin (monoclonal antibody GTU-88, Sigma), cdk5rap2 (rabbit antibody 46024, raised against GST-tagged fusion protein, containing amino acids 1–247 of cdk5rap2), ninein (rabbit antibody 1732, raised against GST-tagged ninein fusion protein), alpha-tubulin (monoclonal antibody DM1a, Sigma), Ki-67 (polyclonal antibody M-19, Santa Cruz Biotechnology), embryonic myosin (monoclonal antibody, developed by Helen Blau, Developmental Studies Hybridoma Bank, University of Iowa), myogenin (monoclonal antibody F5D, Santa Cruz Biotechnology), nuclear pore complex proteins (monoclonal antibody Mab414, Covance), nesprin 1 (rabbit antibody, Abcam). The rabbit antibodies against cdk5rap2 and ninein were raised in our laboratory, and tested for specificity by immunoblotting of bacterial fusion protein and by immunofluorescence in cultured cells. The specificity was further confirmed in experiments using siRNA against cdk5rap2 or ninein, leading to disappearance of the respective immunofluorescence signal (unpublished results). DNA was stained with 4',6-diamidino-2-phenylindole (DAPI). Histological sections of muscle were prepared from pieces of mouse hind leg muscle, embedded and frozen in Tissue-Tek (Sakura), using a Leica cryostat. Sections were subsequently fixed in methanol at -20°C and processed for immunofluorescence.

Immunoelectron microscopy was performed on differentiated C2C12 cells that were fixed overnight in 4% paraformaldehyde in 0.2 M sodium phosphate buffer, pH 7.4. Ultra-thin frozen sections were prepared as described [20], and labelled with rabbit antibody against PCM1, followed by protein A conjugated with 10 nm gold.

### Biochemical methods

C2C12 cells were differentiated for four days, to obtain a high yield of differentiated, fused myotubes. After removal of culture medium, cells were treated with 1× Trypsin/EDTA (Gibco) for several seconds, leading to selective detachment of fused myotubes. Detachment was monitored by phase contrast microscopy. Detached cells were collected and trypsin was neutralized by addition of growth medium. Following centrifugation and two washes with PBS (phosphate-buffered saline), cells were re-suspended in buffer containing 0.2 M KCl, 0.1 M PIPES pH 7.4, 0.2 M MgCl_2_, 10 μM cytochalasin B, 0.1 mM phenylmethyl sulfonylfluoride, and 10 μg/ml of leupeptin, pepstatin, and chymostatin. After an additional centrifugation and wash in the same buffer, cells were homogenized in a douncer with tight-fitting pestle. The material was layered onto a 30% sucrose cushion and centrifuged at 850 × g for 10 minutes. The pellet, containing nuclei, was re-suspended in the same buffer as used for homogenization, and centrifuged for 450 × g for 5 minutes. The resulting nuclei were stored in 50% glycerol, 250 mM sucrose, 80 mM KCl, 20 mM NaCl, 5 mM EGTA, 15 mM PIPES pH 7.4, 1 mM dithiothreitol, 0.5 mM spermidin, 0.2 mM spermin, 0.1 mM phenylmethyl sulfonylfluoride, and 10 μg/ml of leupeptin, pepstatin, and chymostatin. To examine the solubility of perinuclear PCM-1, extraction of nuclei was performed for 10 minutes in the same buffer without glycerol, containing 0.1% Triton X-100, or 1% Triton X-100, or 1.5 M NaCl, or 6 M urea, or 8 M urea, or 2% sodium dodecyl sulfate. Subsequently, immunofluorescence was performed, following centrifugation of extracted nuclei through a cushion of 30% glycerol onto glass coverslips. Cell cycle experiments were performed by incubation of purified nuclei in cytostatic factor-arrested extracts from Xenopus laevis eggs that were prepared as described [21]. Extracts were stimulated to cycle through S-phase by addition of 0.4 mM CaCl_2 _and incubation for approximately 40 to 60 minutes. The passage through S-phase was verified in parallel experiments by monitoring DNA synthesis, by incorporating bromodeoxyuridine into nuclei, followed by immunofluorescence [22]. After 90 minutes, extracts had reached prophase, and a new mitotic state was stabilized by addition of a fresh 50% volume-equivalent of cytostatic factor-arrested extract. Immunofluorescence of nuclei was performed following centrifugation onto glass coverslips as described above. The cell cycle state of extracts was monitored in parallel samples incubated with Xenopus laevis sperm and rhodamine-labelled tubulin, to monitor chromosome condensation and spindle formation.

## Authors' contributions

V.S. and X. F. contributed equally to this manuscript, by participating in the conception of this work, and by performing microscopy and biochemical experiments. C.R. performed electron microscopy experiments. R.H. and A.M. performed experiments on Xenopus egg extracts. A.M. coordinated this study and drafted the manuscript. All authors read and approved the final manuscript.
